# Identification of prostate cancer bone metastasis related genes and potential therapy targets by bioinformatics and in vitro experiments

**DOI:** 10.1111/jcmm.18511

**Published:** 2024-08-04

**Authors:** Haiyang Jiang, Mingcheng Liu, Yingfei Deng, Chongjian Zhang, Longguo Dai, Bingyu Zhu, Yitian Ou, Yong Zhu, Chen Hu, Libo Yang, Jun Li, Yu Bai, Delin Yang

**Affiliations:** ^1^ Department of Urology I The Third Affiliated Hospital of Kunming Medical University (Peking University Cancer Hospital Yunnan, Yunnan Cancer Hospital, Cancer Center of Yunnan Province) Kunming Yunnan China; ^2^ Department of Urology II The second Affiliated Hospital of Kunming Medical University Kunming Yunnan China; ^3^ Department of Human Cell Biology and Genetics, School of Medicine Southern University of Science and Technology Shenzhen China; ^4^ Pathology‐Department The Third Affiliated Hospital of Kunming Medical University (Peking University Cancer Hospital Yunnan, Yunnan Cancer Hospital, Cancer Center of Yunnan Province) Kunming Yunnan China

**Keywords:** bone metastasis, cell communication, hub genes, machine learning, prostate cancer, single cell analysis

## Abstract

The aetiology of bone metastasis in prostate cancer (PCa) remains unclear. This study aims to identify hub genes involved in this process. We utilized machine learning, GO, KEGG, GSEA, Single‐cell analysis, ROC methods to identify hub genes for bone metastasis in PCa using the TCGA and GEO databases. Potential drugs targeting these genes were identified. We validated these results using 16 specimens from patients with PCa and analysed the relationship between the hub genes and clinical features. The impact of *APOC1* on PCa was assessed through in vitro experiments. Seven hub genes with AUC values of 0.727–0.926 were identified. *APOC1, CFH, NUSAP1* and *LGALS1* were highly expressed in bone metastasis tissues, while *NR4A2, ADRB2* and *ZNF331* exhibited an opposite trend. Immunohistochemistry further confirmed these results. The oxidative phosphorylation pathway was significantly enriched by the identified genes. Aflatoxin B1, benzo(a)pyrene, cyclosporine were identified as potential drugs. *APOC1* expression was correlated with clinical features of PCa metastasis. Silencing *APOC1* significantly inhibited PCa cell proliferation, clonality, and migration in vitro. This study identified 7 hub genes that potentially facilitate bone metastasis in PCa through mitochondrial metabolic reprogramming. *APOC1* emerged as a promising therapeutic target and prognostic marker for PCa with bone metastasis.

## 1 INTRODUCTION

Prostate cancer (PCa) has emerged as the most prevalent malignancy in males and the second leading cause of cancer‐related deaths among men.^1^ Although localized PCa is often curable, the number of patients initially diagnosed at a metastatic stage has increased significantly in the last decade.^2^ Metastasis, particularly in the bones, represents a major cause of mortality in patients with PCa,^3^ affecting approximately 70%–90% of those with advanced disease. While novel endocrine therapies combined with paclitaxel chemotherapy have shown promising results, resistance to these drugs often develops, rapidly leading to metastatic castration‐resistant PCa(mCRPC) and making treatments ineffective.^4^, ^5^, ^6^ Patients with bone metastases are at increased risk of fractures, severe bone pain, and paraplegia, which greatly diminish the quality of life and worsen prognosis.^7^, ^8^

Previous studies have demonstrated that the process of bone metastasis in Pca is a complex and sequential cascade involving various cellular interactions within the tumour microenvironment.^8^, ^9^ Within this intricate process lies the potential influence of certain small RNAs on gene expression modulation and disease progression. In recent years, exosomes as well as circRNAs and microRNAs have emerged as key players contributing positively to cancer metastasis dynamics including invasion mechanisms and drug resistance.^10^, ^11^, ^12^ Given the limited therapeutic options for PCa with bone metastasis, understanding the underlying molecular mechanisms holds immense clinical significance for improving patient outcomes.

Integrating scRNA‐seq with bulk RNA‐seq allows for a more detailed and accurate depiction of gene expression, aiding researchers unravel biological functions and mechanisms of key molecules.^13^, ^14^, ^15^ Machine learning (ML) draws on techniques from statistics, computer science, and artificial intelligence (AI) to provide new solutions for uncovering complex relationships and patterns that traditional statistical methods may not be able to elucidate, thereby helping in the development of more efficient algorithms.^16^ Recently, bioinformatics and ML have enabled a more comprehensive and in‐depth analysis of multi‐omics data.^17^, ^18^, ^19^

**FIGURE 1:**
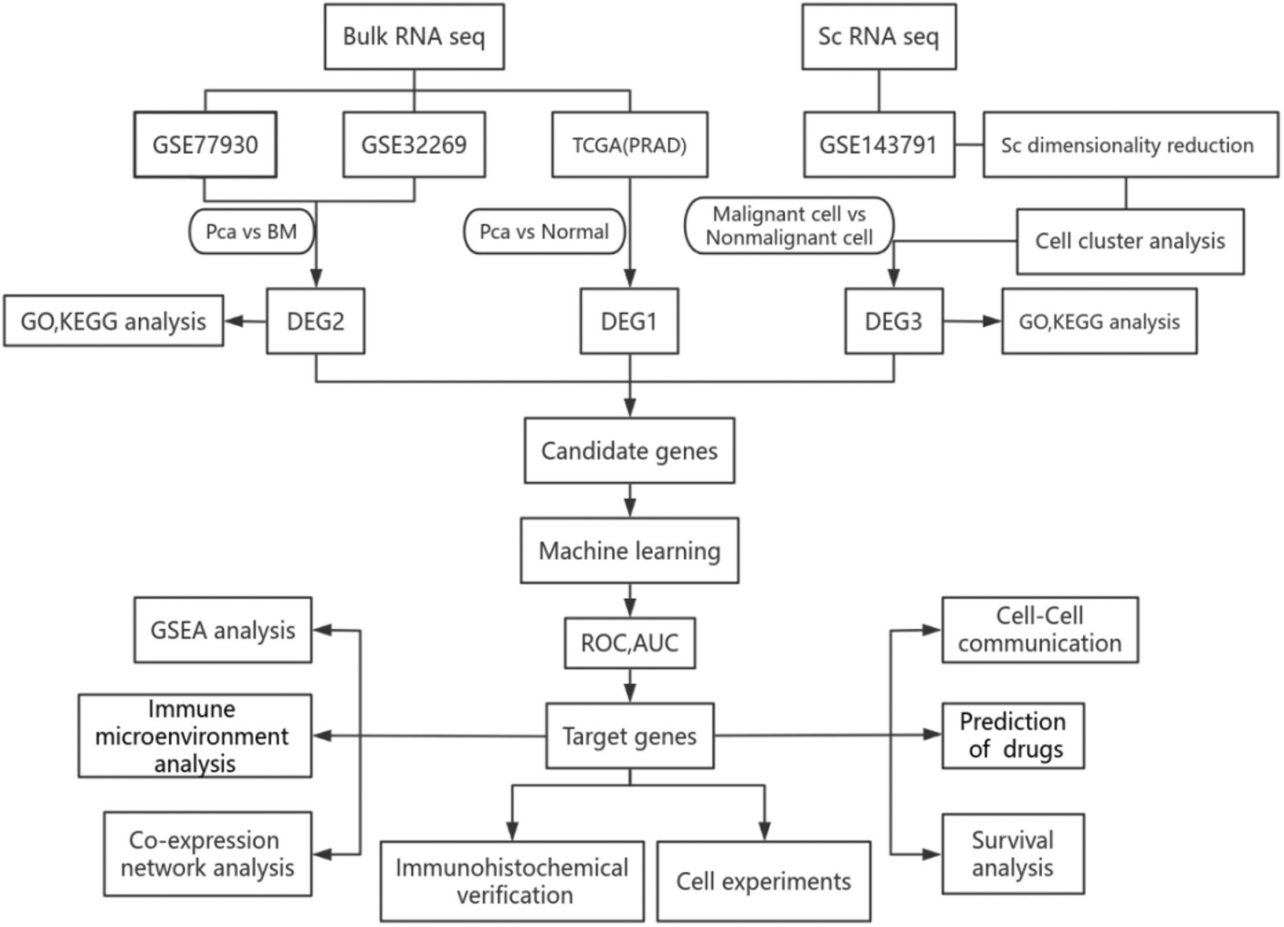
Flow chart. PCa (prostate cancer), BM (bone metastasis in prostate cancer).

In this study, we employed a series of bioinformatics methods, including ML, differential expression, enrichment, co‐expression network, immune infiltration, and intercellular communication analyses to identify hub genes involved in PCa with bone metastasis. These genes were subsequently validated using pathological tissue sections and clinical data from patients with PCa. Finally, the role of APOC1 in PCa cells was confirmed through in vitro experiments. The flowchart of this study is shown in Figure 1.

## 2 EXPERIMENTAL PROCEDURES

### 2.1 Data collection

TCGA‐PRAD was downloaded from The Cancer Genome Atlas (TCGA) database. After eliminating duplicate patient samples, 532 samples (51 normal and 481 prostate adenocarcinoma (PRAD) samples) were obtained.

PCa‐related transcriptome datasets (GSE77930 and GSE32269) and a scRNA‐seq dataset (GSE143791) were all acquired from the Gene Expression Omnibus (GEO) database. GSE77930 (GPL15659) included 22 PCa and 20 PCa with bone metastasis samples. GSE32269 (Affymetrix Human Genome U133A Array) comprised 51 samples, including 22 PCa and 29 PCa with bone metastasis samples. The GSE143791 dataset included 9 PCa with bone metastases and 7 normal samples.

Pathological tissue specimens were collected from 16 patients diagnosed with PCa and bone metastasis between 2019 and 2023. All patients were recruited from The Third Affiliated Hospital of Kunming Medical University (Peking University Cancer Hospital Yunnan, Yunnan Cancer Hospital, Cancer Center of Yunnan Province). Case information is provided in Table 1.

**TABLE 1:** Details of the cases used in this study.

Characteristics				
Age	<65	65–70	>70	
*n*	5	7	4	
Gleason	G(7)	G(8)	G(9)	
*n*	4	8	4	
ADT therapy	Goserelin	Leuprorelin		No therapy
*n*	5	7		4
Endocrine therapy	Bicalutamide	Abiraterone	Apalutamide	No therapy
*n*	4	5	3	4

### 2.2 Differentially expressed gene analysis

Differentially expressed genes 1 (DEGs1) between PRAD and normal groups in the TCGA‐PRAD dataset were screened by DESeq2 package.^20^ The limma package^21^ was used to identify candidate differentially expressed genes 2 (DEGs2) between PCa and PCa with bone metastasis in GSE77930 and GSE32269 datasets. The screening criteria were both |log2FoldChange (FC)| >0.5 and adjusted (adj.) *p* < 0.05. Findmarker function was used to identify genes specifically related to PCa with bone metastasis from the GSE143791 dataset. Genes differentially expressed 3 (DEGs3) between tumour and non‐tumour cells in bone metastases were assessed using a threshold of average log FC = 0.5 and adj.*p* < 0.05.

### 2.3 Identification and function analyses of DEGs

After that, GO and KEGG enrichment analyses were performed to identify the functions and pathways affected by DEGs 2, DEGs 3 using the ClusterProfiler package.^22^

### 2.4 Screening of hub genes

Cadidate genes associated with bone metastasis PCa were identified by intersecting DEGs1, DEGs2 and DEGs3. Two machine‐learning algorithms (Least Absolute Shrinkage and Selection Operator [LASSO] and Support Vector Machine–Recursive Feature Elimination [SVM‐RFE]) were used to identify signature genes in the GSE77930 dataset. The common signature genes derived from both algorithms were identified as hub genes. The diagnostic efficacy of these genes in GSE77930 and GSE32269 was assessed using Receiver Operating Characteristic (ROC) curves generated with the pROC package.^23^ Expression trends of hub genes in these datasets were visualized using the ggplot2 package.^24^

### 2.5 Immunohistochemistry

Paraffin sections were placed in an 60°C for 2 h, dewaxed with xylene and ethanol, and rinsed three times with distilled water and phosphate‐buffered saline (PBS) to ensure tissue integrity. Subsequently, the slides were immersed in 3% hydrogen peroxide at 37°C for 5 min to inhibit endogenous peroxidase enzymes, while avoiding light exposure. Next, the slides were incubated overnight at 4°C with anti‐*AOPC1*, anti‐*CFH*, anti‐*NR4A2*, anti‐*NUSAP1* primary antibodies. The next day, the slides were incubated with secondary antibodies at 37°C for 1 h, followed by three PBS finses, each lasting 5 min. Diaminobenzidine tetrahydrochloride (DAB) was added until satisfactory staining, and the slides were rinsed with purified water. Finally, slides were stained with haematoxylin and covered with coverslips, and the immunohistochemistry (IHC) scores were determined by multiplying two scores: ① staining intensity: 0 (negative), 1 (weak positive), 2 (moderate positive), 3 (strong positive); ② percentage of positive cells: 0 (<5%), 1 (5%–25%), 2 (26%–50%), 3 (51%–75%), 4 (>75%).^25^

### 2.6 Gene set enrichment analysis (GSEA) and co‐expression network of hub genes

To explore corresponding pathways and potential biological mechanisms, the clusterProfiler package^26^ and org.Hs.eg.db packages^27^ were used for enrichment analysis of hub genes. According to correlations between hub genes and the expression levels of each gene in the GSE77930 dataset, all genes were ranked. GSEA was conducted with the criteria of |NES| >1, NOM *p* < 0.05 and *q* < 0.25. Furthermore, a co‐expression network of hub genes was constructed by GeneMANIA database.

### 2.7 Immune infiltration analysis and drug prediction

Differences in immune cells infiltration between PCa and PCa with bone metastasis were compared by Wilcoxon test. Spearman correlations were used to analyse the relationships between differential immune cells and between differential immune cells and hub genes. The correlation diagram was created using the ggcorrplot package.^28^ Additionally, the Comparative Toxicogenomics Database (CTD) was used to identify drugs potentially targeting hub genes for the treating PCa with bone metastasis. Key drugs were identified by intersecting the predictions for all hub genes. The visualization of the drugs‐hub genes network was accomplished using the Cytoscape package.^29^

### 2.8 Single cell analysis

The single‐cell data from the GSE143791 dataset was filtered by seurat package^30^ with following criteria: (1) Genes detected in fewer 200 cells were removed. (2) Cells with ≤200 genes and ≥5000 genes were excluded. (3) Cells with a unique molecular identifier (UMI) count below 1000 and those in the top 3% of the count ranking were removed. (4) Cells with less than 10% mitochondrial genes were retained. The IntegrateData function merged data of 16 samples from GSE143791. After normalization, highly variable genes underwent variance stabilization transformation. Subsequently, principal components analysis was conducted on these genes for dimensional reduction of the scRNA‐seq data. The FindNeighbors and FindClusters functions in seurat package were used to carry out unsupervised cell cluster analysis. t‐distributed Stochastic Neighbour Embedding (tSNE) and Uniform Manifold Approximation and Projection (UMAP) were used for visualizing high‐dimensional data. Cell type were annotated based on the annotation file of the raw dataset, and the distribution of each cell type was compared between PCa with bone metastasis and normal samples using a t‐test.

The expression of hub genes in annotated cells was visualized. To understand cell interaction differences between PCa with bone metastasis and PCa, cellular communication analysis was performed using the CellChat package.^31^ The possible interactions among cells were evaluated based on ligand‐receptor pairs, leading to the construction of a ligand‐receptor network.

### 2.9 Analysis of the relationship between hub genes and clinical characteristics of PCa

The TCGA‐PRAD dataset included 553 patients, with 10 deaths, five of which were specifically from PCa. Due to insufficient data to analyse the relationship between overall survival (OS), disease‐free survival (DFS), and bone metastasis‐specific death, our focus was on the relationship between hub genes and Progression Free Interval (PFI). Using the ‘survival’, ‘survminer’, and ‘ggplot2’ packages, we analysed the correlation between the seven hub genes and PFI in the TCGA‐PRAD dataset. Additionally, we utilized the ‘ggplot2’, ‘stats’, and ‘car’ packages to investigate the association between APOC1 expression and the clinical characteristics of PCa, such as pathological stage, primary therapy outcome, residual tumour presence, Gleason score, and PFI.

### 2.10 Cell culture

The human PCa cell lines C4‐2B and PC‐3 were cultured at 37°C with 5% CO2. Subsequently, they were transfected with the siRNA sequence: 5′‐UGAACUUUCUGCCAAGAUG‐3′ (RIBOBIO). After 4–6 h of transfection, the medium was replaced with fresh complete medium.

### 2.11 RT‐PCR

Using an SYBR green qPCR kit (TransGen) according to the manufacturer's instructions, we performed Real‐time PCR to detect *APOC1* transcription products. We utilized β‐actin as the internal controls. The primers were: *APOC1* gene forward primer: 5′‐TCCAGTGCCTTGGATAAGCTG‐3′ and reverse primer: 5′‐GGCTGATGAGTTCCCGAGC‐3′; β‐actin gene forward primer: 5′‐GTGGCCGAGGACTTTGATTG‐3′ and reverse primer: 5′‐CCTGT AACAACGCATCTCATATT‐3′. The mRNA expression levels were quantified using the 2^−ΔΔCq^ method.^32^

### 2.12 Cell viability assessment

The PCa cell line C4‐2B was cultured in 96‐well plates and transfected with corresponding si‐RNAs. Cell viability was assessed using the Cell Counting Kit‐8 (CCK‐8) (Beyotime Biotechnology). After adding the CCK‐8 reagent, the cells were incubated at 37°C for 1.5 h. Viability was measured at 24, 48 and 72 h using an ELISA kit by reading the absorbance at 450 nm.

### 2.13 Experiment on soft agar colony formation

1000 C4‐2B cells/well and 500 PC‐3 cells/well were seeded into 6‐well plates, and cultured for 14 days to form clonal colonies. These colonies were fixed with 4% paraformaldehyde, stained with 0.05% crystal violet for 1 h at room temperature, and then photographed.

### 2.14 Transwell assays

Approximately 5 × 10^5^ C4‐2B cells transfected with the corresponding siRNA were seeded in the top chamber of a polycarbonate Transwell chamber and cultured at 37°C and with 5% CO_2_ for 48 h. The cells were fixed with 5% glutaraldehyde, stained with 0.05% crystal violet, and counted under a microscope.

### 2.15 Cell scratch assay

C4‐2B, PC3 cells transfected with si‐RNA or si‐NC were inoculated on a 6‐well plate at a density of 1 × 10^5^ cells per well. Use the tip of a 200 uL pipette to make scratches on the monolayer of cells. Wash the samples with PBS to remove cell debris. Next, the cells were cultured for 48 h, observed and photographed at 0 and 48 h.

### 2.16 Statistical analysis

The R software was used to process and analyse the data. The *p* < 0.05 were considered statistically significant.

## 3 RESULTS

### 3.1 Identification of DEGs1 and DEGs2

**FIGURE 2:**
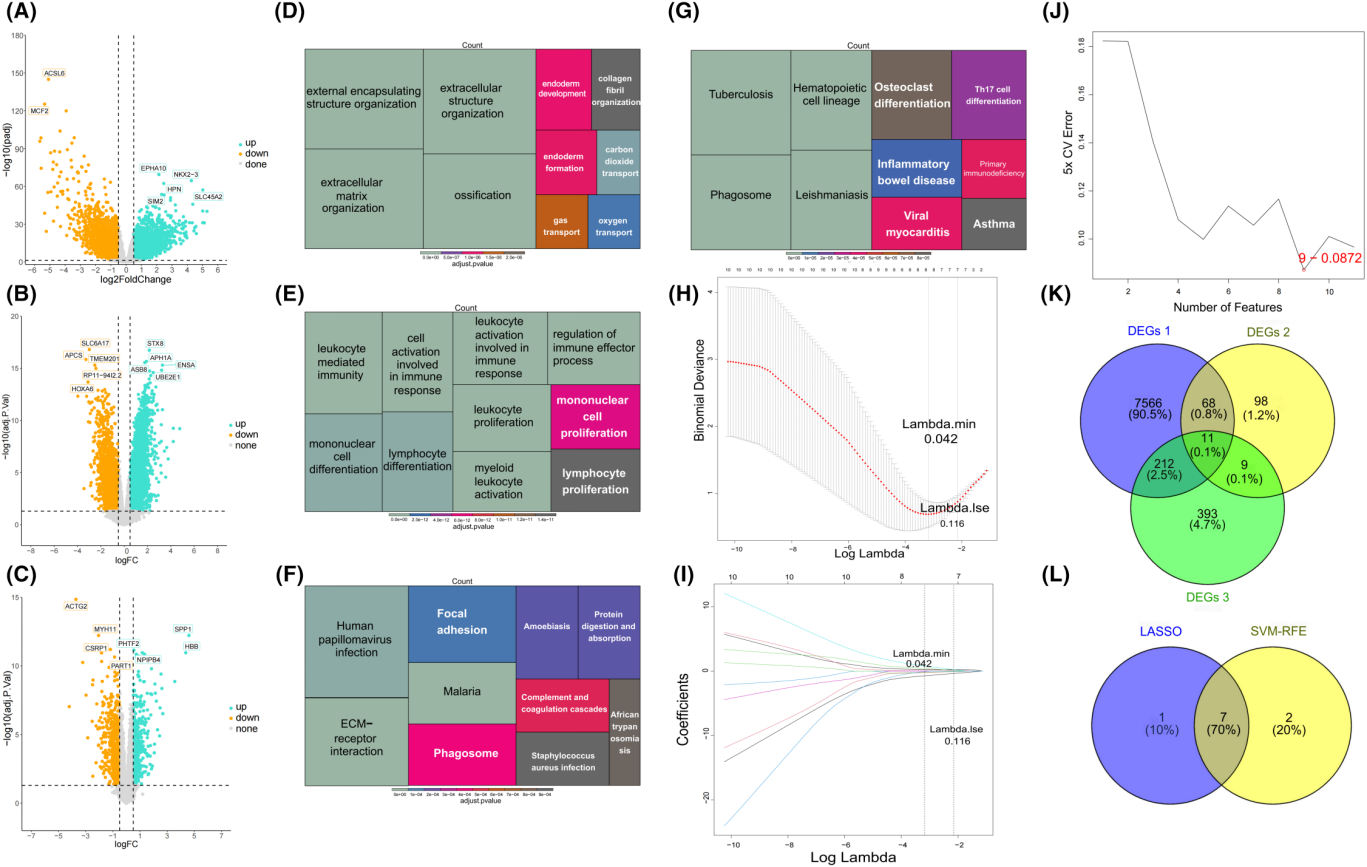
Differential gene expression analysis, enrichment analysis and identification of potential genes associated with bone metastasis in PCa. (A–C) Volcano plot illustrating the differential expression of genes. (D) DEGs2 GO enrichment analysis. (E) DEGs2 KEGG enrichment analysis. (F) DEGs3 GO enrichment analysis. (G) DEGs3 KEGG enrichment analysis. (H, I) LASSO regression of 11 genes. (J) SVM‐RFE analysis. (K) DEGs 1,2,3 Venn diagram illustrating candidate genes. (L) Intersection of two machine learning feature genes.

Differential expression analysis identified 7857 DEGs 1 between PRAD and normal groups in the TCGA‐PRAD dataset, with 4039 upregulated and 3818 downregulated genes (Figure 2A; Figure S1A). In the GSE77930 datasets, we found 7166 candidate genes between PCa with bone metastasis and PCa, consisting of 4096 upregulated and 3070 downregulated genes, and 1163 candidate genes, with 521 upregulated and 651 downregulated genes in the GSE32269 datasets (Figure 2B,C; Figure S1B,C).

By intersecting candidate genes in the GSE77930 and GSE32269 datasets, we identified 186 DEGs 2 (Figure S1D,E). The analysis of DEGs 2 revealed enrichment in 168 GO terms, including 98 biological processes (BPs), 39 cellular components (CCs), and 31 molecular functions (MFs). The most prominent enrichment was found in terms related to extracellular matrix organization, extracellular structure organization, collagen‐containing extracellular matrix, endoplasmic reticulum lumen, extracellular matrix structural constituent, and extracellular matrix binding (Figure 2D). Additionally, 12 KEGG pathways were enriched by DEGs2 including extracellular matrix (ECM)‐receptor interaction, malaria, and amoebiasis (Figure 2E).

### 3.2 Identification and functional analysis of DEGs3

We identified 625 DEGs3 between tumour and non‐tumour cells in PCa with bone metastasis. These genes were then analysed using GO and KEGG enrichment analyses to understand their potential functions and associated pathways (Table S1). Results showed 903 GO terms and 38 KEGG pathways were enriched by DEGs3. For BP, terms related to leukocyte activation involved in immune response and myeloid leukocyte activation were predominant. For CC such as secretory granule lumen and vesicle lumen were significantly enriched. In MF, the most enriched terms included immune receptor activity, cell adhesion mediator activity, major histocompatibility complex (MHC) protein complex binding (Figure 2F). Key enriched KEGG pathways included leishmaniasis, haematopoietic cell lineage, tuberculosis, phagosome, inflammatory bowel disease (Figure 2G).

### 3.3 APOC1, LGALS1, CFH, NUSAP1, NR4A2, ADRB2 and ZNF331 were identified as hub genes

Totally 11 candidate genes were identified by overlapping 7857 DEGs1, 186 DEGs2 and 625 DEGs3 (Figure 2K). By LASSO algorithm, 8 signature genes were selected at λ min = 0.042 (Figure 2H,I). Subsequently, the SVM‐RFE algorithm was applied to identify an optimal combination of nine signature genes (minimal RMSE = 0.0872) were finally obtained (Figure 2J). Ultimately, *APOC1, LGALS1, CFH, NUSAP1, NR4A2, ADRB2* and *ZNF331* were identified as hub genes by overlapping the signature genes from both machine‐learning algorithms (Figure 2L).

### 3.4 Verification and expression analyses of hub genes

**FIGURE 3:**
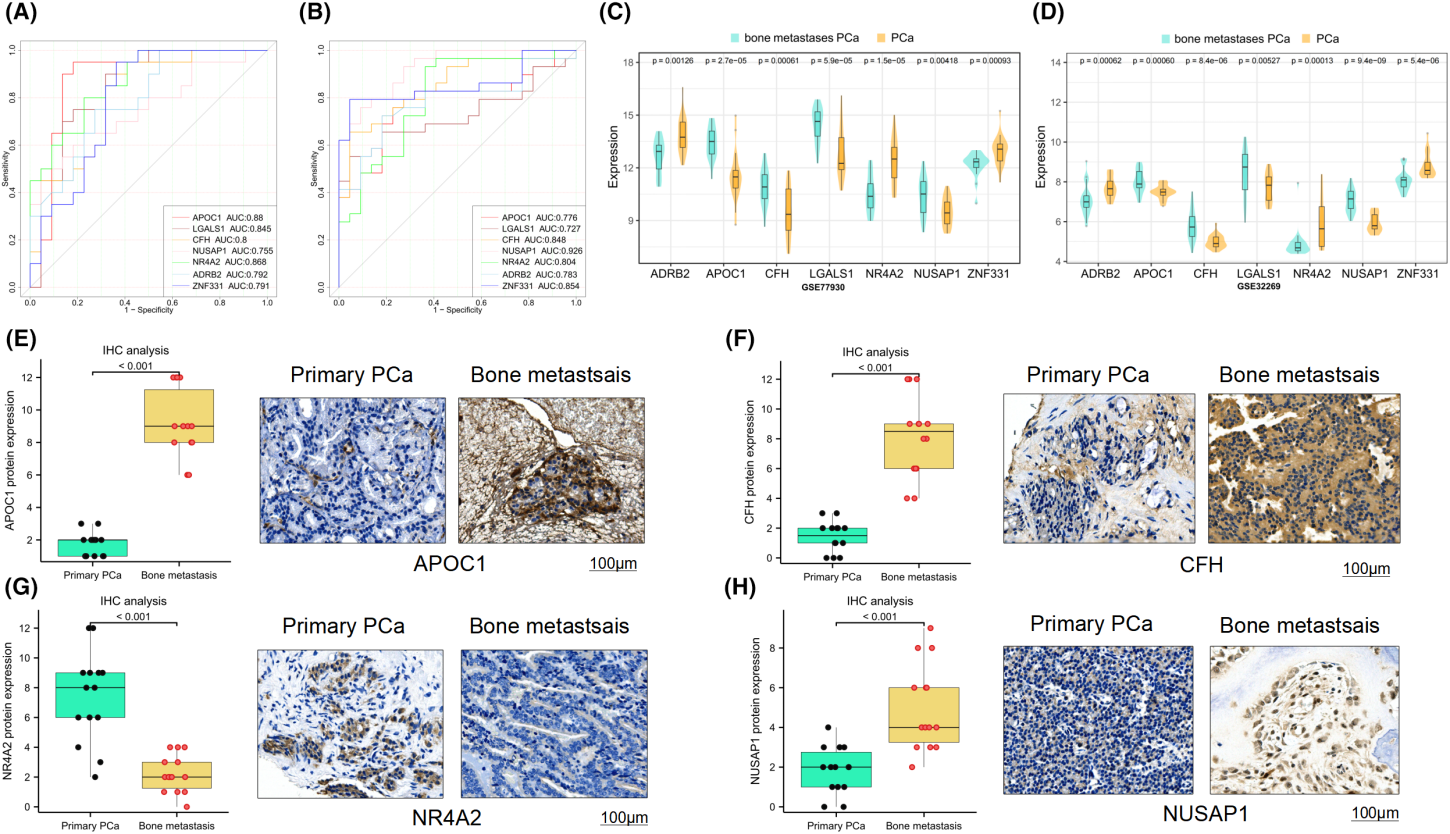
Critical genetic diagnostic efficacy and Revalidation of genes expression. (A) ROC Curve Analysis of Key Genes in GSE77930. (B) ROC Curve Analysis of Key Genes in GSE32269. (C) Verification of key genes expression in GSE77930. (D) Verification of key genes expression in GSE77930 in GSE32269. (E) Differential expression of APOC1 protein in primary PCa tissues, bone metastasis tissues and representative images of immunohistochemistry. (F) Differential expression of CFH protein in primary PCa tissues, bone metastasis tissues and representative images of immunohistochemistry. (G) Differential expression of NR4A2 protein in primary PCa tissues, bone metastasis tissues and representative images of immunohistochemistry. (H) Differential expression of NUSAP1 protein in primary PCa tissues, bone metastasis tissues and representative images of immunohistochemistry.

The AUC values of hub genes in the GSE77930 and GSE32269 datasets exceeded 0.7, indicating good diagnostic efficacy (Figure 3A,B). Furthermore, the expression of these genes differed significantly between PCa with bone metastasis and PCa groups, exhibiting consistent trends. *ADRB2, NR4A2* and *ZNF331* had higher levels of expression in PCa than in PCa with bone metastasis groups, while the remaining 4 hub genes exhibited opposite trends (*p* < 0.05) (Figure 3C,D). In addition, immunohistochemical analysis further confirmed that *APOC1, CFH, NR4A2* and *NUSAP1* expression at the protein level was higher in primary PCa tissues than in PCa with bone metastasis tissues (*p* < 0.05) (Figure 3E,F,H), while *NR4A2* exhibited the opposite trend (*p* < 0.05) (Figure 3G). These results were consistent with the results of the bioinformatics analysis.

### 3.5 Common pathways and genes enriched by hub genes

**FIGURE 4:**
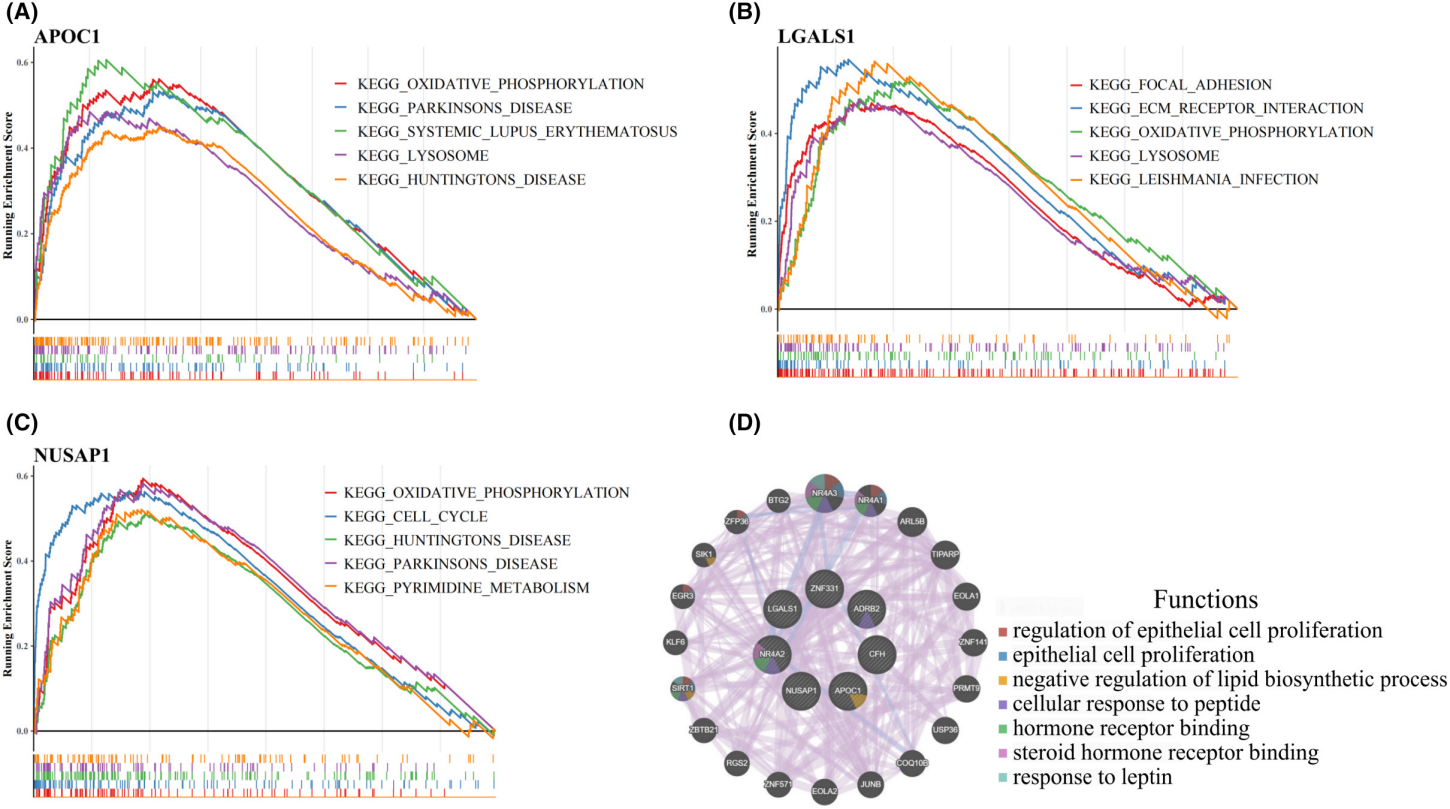
GSEA enrichment analysis of hub genes and reconstruction of co‐expression networks. (A–C) GSEA analysis of *APOC1, LGALS1* and *NR4A2*. (D) Co‐expression network construction.

Based on GSEA, 47, 50, 57, 27, 22, 36 and 23 KEGG pathways were enriched by *APOC1, LGALS1, CFH, NUSAP1, NR4A2, ADRB2* and *ZNF331* respectively. Top 5 significantly enriched pathways for each hub genes are shown in Figure 4A–C; Figure S2A–D. Oxidative phosphorylation was enriched by *APOC1, LGALS1, NUSAP1, NR4A2, ADRB2* and *ZNF331*. Parkinson's and Huntington's disease were both enriched by *APOC1, NUSAP1, NR4A2, ADRB2* and *ZNF331*. Additionally, a gene interaction network was constructed around the hub genes with 20 neighbouring genes. *NR4A3* and *NR4A1* were the genes exhibiting the highest correlations. The primary functions involved were epithelial cell proliferation and negative regulation of lipid biosynthetic processes (Figure 4D).

### 3.6 PCa with bone metastasis exhibited a different immune microenvironment

**FIGURE 5:**
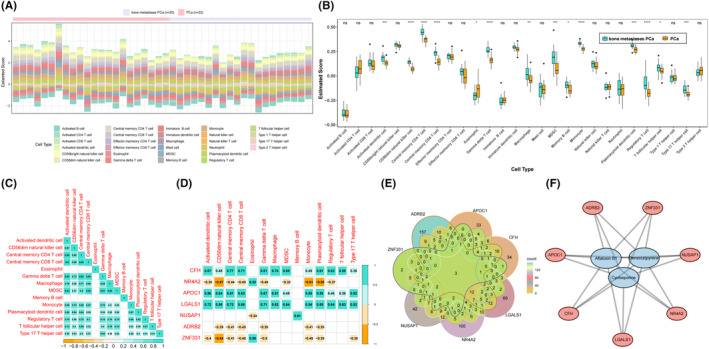
Analysis of tumour microenvironment and drug predictors. (A) Heat map of immune cells infiltration. (B) Box plot illustrating variations in immune cells infiltration. (C) Diagram illustrating the correlation between different immune cell types. (D) Correlation between key genes and differential immune cells. (E) Venn diagram of key genes drug predictors. (F) Network diagram for predicting key genes and drug interactions.

The distribution of 28 immune cell types is illustrated in Figure 5A. 14 of these exhibited significant differences between PCa with and without bone metastasis, except for eosinophils, all others were more prevalent in PCa with bone metastasis than in PCa (Figure 5B). The correlation analysis revealed strong positive correlations between central memory CD4 T, gamma delta T and myeloid‐derived suppressor cells with activated dendritic cells (*p* < 0.05, *r* > 0.8) (Figure 5C). As shown in Figure 5D, *CFH, APOC1* and *LGALS1* were positively correlated with the most differential immune cells (*p* < 0.05, *r* > 0.5). Conversely, *NR4A2, NUSAP1, ADRB2* and *ZNF331* were negatively correlated with differential immune cells such as CD56dim natural killer cells, monocytes and plasmacytoid dendritic cells (*p* < 0.05, *r* < −0.5). Moreover, drug prediction for the hub genes identified aflatoxin B1, benzo(a)pyrene and cyclosporine as drugs targeting all seven hub genes simultaneously (Figure 5E,F).

### 3.7 Hub genes were performed single cell communication

After filtering out low‐quality data (Figure S3), 2000 highly variable genes were selected for further analysis, with the top 15 genes highlighted in Figure S4A. The top 30 dimensions were used for tSNE and UMAP analyses (Figure S4B). Subsequently, 28 distinct cell subpopulations were annotated (Figure S4C). Of these, 11 types showed differential distribution: cells such as resting regulatory T (Treg) cells, Cytotoxic T lymphocytes (CTL)‐1, CTL‐2, and Mono2 were more prevalent in the normal group, while oteoblasts and tumour‐associated macrophages (TAM) were more common in PCa with bone metastasis group (Figure S5).

Expression of hub genes across 28 cell subpopulations is shown in Figure S6. *ADRB2* and *APOC1* exhibited significant differences in 16 cell types between PCa with bone metastasis and PCa. *LGALS1* and *NUSAP1* differed in 11 cell types, *CFH* and *NR4A2* in 10 and *ZNF331* in 9.

Cellular communication analysis was carried out to understand the interactions between PCa with bone metastasis and normal groups. Results indicated significant changes in interaction strength. Communication between osteoblasts, pericytes, and endothelial cells was more frequent and stronger in PCa with bone metastasis (Figure S4D) than in the normal group (Figure S4E).

### 3.8 Relationship between hub genes and clinical features

**FIGURE 6:**
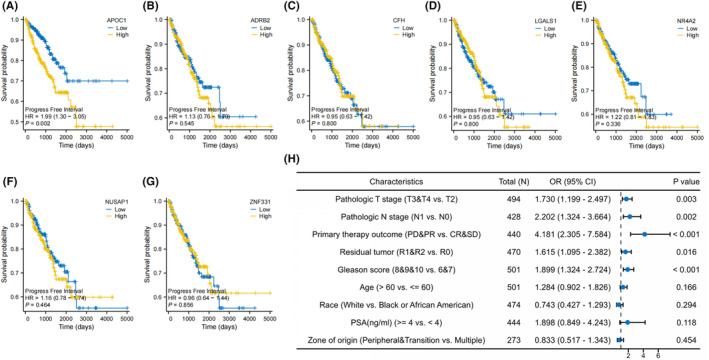
Correlation analysis between *APOC1* and clinical features based on TCGA database. (A) The predictive value of *APOC1* for PFI in prostate cancer. (B) The predictive value of *ADRB2* for PFI in prostate cancer. (C) The predictive value of *CFH* for PFI in prostate cancer. (D) The predictive value of *LGALS1* for PFI in prostate cancer. (E) The predictive value of *NR4A2* for PFI in prostate cancer. (F) The predictive value of *NUSAP1* for PFI in prostate cancer. (G) The predictive value of *ZNF331* for PFI in prostate cancer. (H) The forest plot was utilized to demonstrate the risk factors associated with high expression of *APOC1* mRNA, as determined through logistic regression analysis.

**FIGURE 7:**
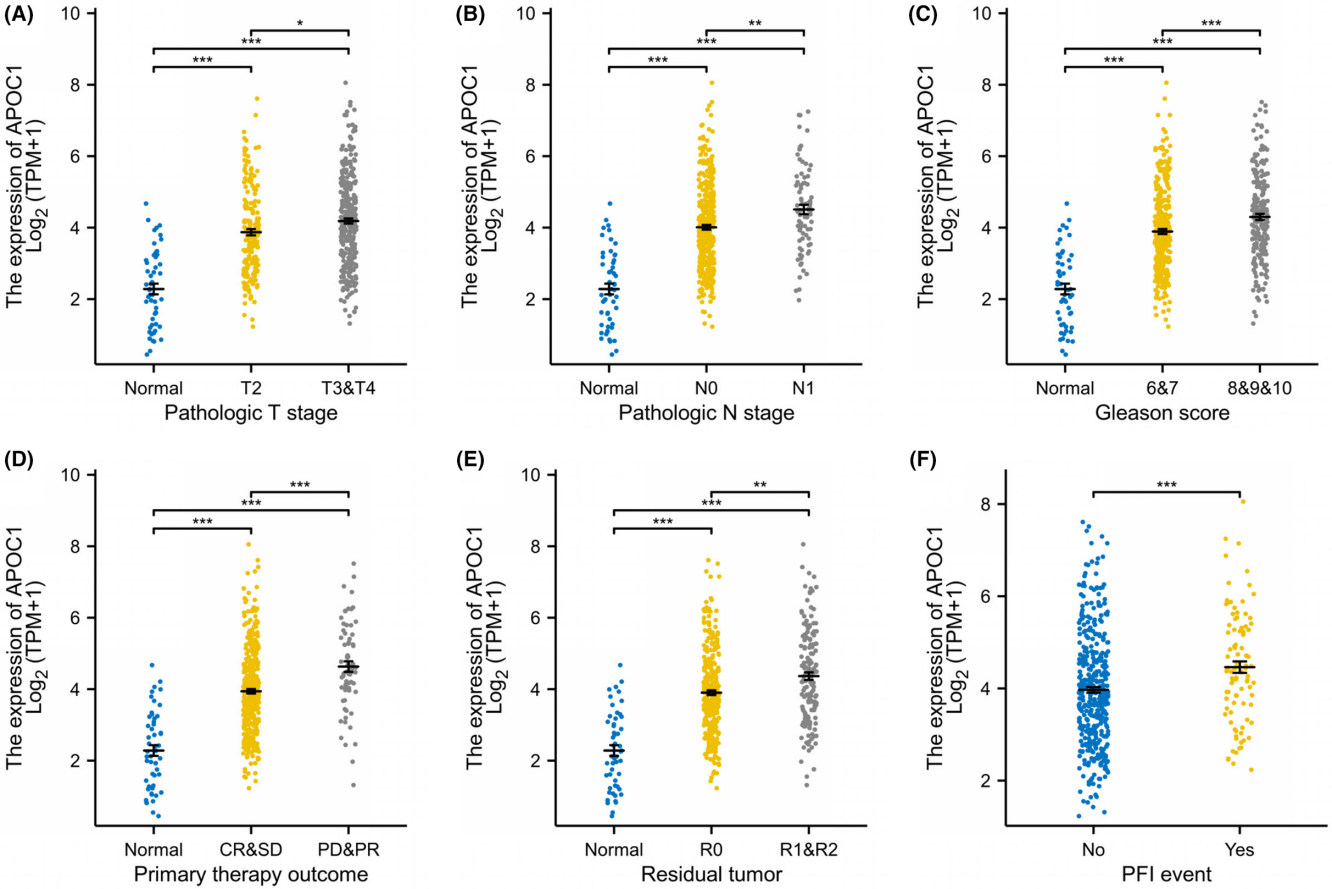
Correlation analysis between *APOC1* and clinical features based on the TCGA database was conducted to investigate the association between mRNA expression of *APOC1* and various clinical characteristics, including: (A) Pathologic T stage. (B) Pathologic N stage. (C) Gleason score. (D) Primary therapy outcome. (E) Residual tumour. (F) PFI event. *p < 0.05, **p < 0.01 and ***p < 0.001.

The available clinical information is summarized in Table 2. Among the seven hub genes, *APOC1* had a significant correlation with PFI (*p* < 0.05) (Figure 6A). Furthermore, *APOC1* expression was higher in stages T3 and T4 than in stage T2 (*p* < 0.01) (Figure 7A) and was greater in patients with lymph node matastasis (N1) compared to those without (N0) (*p* < 0.001) (Figure 7B). Initial treatment outcomes were categorized into complete response (CR), partial response (PR), stable disease (SD), and progressive disease (PD). *APOC1* expression was significantly higher in the PR and PD groups than in the CR and SD groups (*p* < 0.001) (Figure 7D). It was also higher in the Gleason >7 group than in the ≤7 group (*p* < 0.001) (Figure 7C). In terms of residual tumour presence, *APOC1* expression levels were significantly higher in the R1 and R2 groups than in the R0 group (*p* < 0.001) (Figure 7E). Furthermore, the levels of expression of *APOC1* were elevated in patients with disease progression compared to those without (*p* < 0.001) (Figure 7F). Logistic regression analysis identified that pathological stages T3/4, lymph node metastasis (PR/PD), residual tumours (R1/R2), and a Gleason score >7 as risk factors for high *APOC1* expression in PCa (Figure 6H).

**TABLE 2:** Associations between *APOC1* expression and clinical features of patients with PCa in TCGA database.

Characteristics	Low expression of *APOC1*	High expression of *APOC1*	*p*‐Value
*n*	250	251	
Pathologic T stage, *n* (%)			
T2	110 (22.3%)	79 (16%)	0.007
T3	133 (26.9%)	161 (32.6%)	
T4	3 (0.6%)	8 (1.6%)	
Pathologic N stage, *n* (%)			
N0	184 (43%)	164 (38.3%)	0.002
N1	27 (6.3%)	53 (12.4%)	
Clinical T stage, *n* (%)			
T1 & T2	187 (45.9%)	165 (40.5%)	0.040
T3	20 (4.9%)	33 (8.1%)	
T4	0 (0%)	2 (0.5%)	
Clinical M stage, *n* (%)			
M0	226 (49.1%)	231 (50.2%)	0.259
M1	0 (0%)	3 (0.7%)	
Primary therapy outcome, *n* (%)			
CR & SD	207 (47%)	164 (37.3%)	<0.001
PD & PR	16 (3.6%)	53 (12%)	
Age, *n* (%)			
≤60	120 (24%)	105 (21%)	0.165
>60	130 (25.9%)	146 (29.1%)	
Residual tumour, *n* (%)			
R0	171 (36.4%)	145 (30.9%)	0.053
R1	63 (13.4%)	86 (18.3%)	
R2	2 (0.4%)	3 (0.6%)	
Gleason score, *n* (%)			
6 and 7	166 (33.1%)	128 (25.5%)	<0.001
8–10	84 (16.8%)	123 (24.6%)	
PSA (ng/mL), *n* (%)			
<4	220 (49.5%)	197 (44.4%)	0.113
≥4	10 (2.3%)	17 (3.8%)	
OS event, *n* (%)			
Alive	246 (49.1%)	245 (48.9%)	0.754
Dead	4 (0.8%)	6 (1.2%)	
DSS event, *n* (%)			
No	248 (49.7%)	246 (49.3%)	0.996
Yes	2 (0.4%)	3 (0.6%)	
PFI event, *n* (%)			
No	218 (43.5%)	189 (37.7%)	<0.001
Yes	32 (6.4%)	62 (12.4%)	

### 3.9 The effect of APOC1 on the proliferation of PCa cells

**FIGURE 8:**
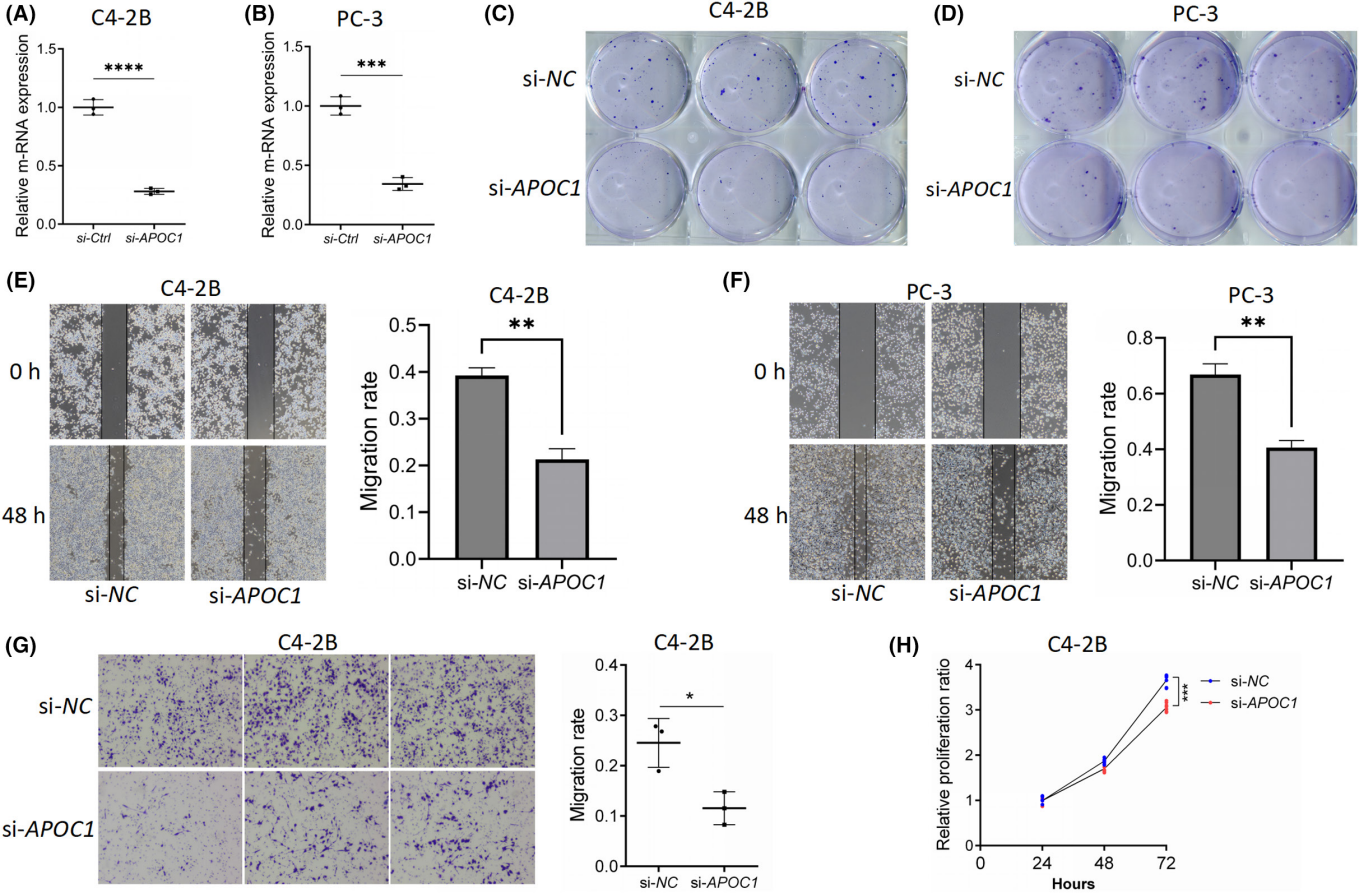
Effects of silencing *APOC1* were detected in vitro. (A, B) RT‐PCR was used to detect the efficiency of *APOC1* down‐regulation in C4‐2B and PC‐3 cell lines. (C, D) Colony formation assay was used to detect the cell viability of C4‐2B and PC‐3 cell lines. (E, F) Cell scratch assay was used to detect the migration ability of C4‐2B and PC‐3 cell lines. (G) Transwell assay was used to detect the migration ability of C4‐2B cell line. (H) CCK‐8 assay was used to detect the proliferation ability of C4‐2B cell line. *p < 0.05, **p < 0.01, ***p < 0.001 and ****p < 0.0001.

To investigate the role of *APOC1* in PCa cells, we conducted in vitro experiments using C4‐2B and PC3 cell lines. RT‐PCR demonstrated a significant down‐regulation of *APOC1* expression in both C4‐2B and PC3 cell lines following si‐*APOC1* transfection (*p* < 0.001) (Figure 8A,B). *APOC1* silencing significantly impaired the colony formation and migration ability of both C4‐2B and PC3 cells (Figure 8C–F). Similarly, transwell assays revealed that silencing *APOC1* silencing decreased the migration and invasion ability of C4‐2B cells (*p* < 0.05) (Figure 8G). After 72 h, the si‐*APOC1* group exhibited significantly reduced C4‐2B cell proliferation compared to the si‐NC group (*p* < 0.05) (Figure 8H).

## 4 DISCUSSION

Bone metastasis significantly worsens the prognosis and survival of patients with PCa, yet its underlying mechanisms are not well understood. This study employed ML techniques to identify key genes associated with bone metastasis in PCa, potentially providing novel therapeutic targets for patient management.

First, we conducted a comprehensive analysis of bulk RNA‐seq and scRNA‐seq datasets from primary PCa and PCa with bone metastasis. Utilizing LASSO and SVM‐REF machine learning methods, we identified seven molecular markers (*ADRB2, APOC1, CFH, LGALS1, NR4A2, NUSAP1* and *ZNF331*) for predicting bone metastasis. Both training and validation sets exhibited high diagnostic performance, with AUC values ranging from 0.727 to 0.926. Furthermore, the expression of these markers was validated in pathological tissues obtained from patients with primary PCa and PCa with bone metastasis. Bioinformatics analysis revealed that *APOC1, CFH, LGALS1* and *NUSAP1* were highly expressed in bone metastases compared to primary tumours, whereas *ADRB2, NR4A2* and *ZNF331* exhibited the opposite trend (*p* < 0.05). Notably, these expression patterns were consistent with our predictions.

*APOC1*, encoding smallest apolipoprotein, has been linked to the progression of PCa. Silencing *APOC1* halts cell cycle progression and enhances apoptosis in PCa cell lines; however, the underlying mechanism remains unclear.^33^, ^34^ Complement factor H (*CFH*) serves as a marker of epithelial‐mesenchymal transition (EMT) in PCa and is linked to cancer‐associated fibroblasts (CAF).^35^ Nucleolar and spindle‐associated protein 1 (*NUSAP1*) participates in the EMT of PCa, promoting its invasion and metastasis.^36^ The β2‐adrenergic receptor (*ADRB2*) plays a role in the neuroendocrinisation of PCa (NEPC). Lower expression levels of ADRB2 are associated with shorter OS rates in patients with PCa.^37^ *LGALS1* encodes galectin‐1. Silencing *LGALS1* inhibits the growth and invasion of mCRPC cells through modulation of the androgen receptor (AR) and AKT pathway.^38^ *NR4A2*, a member of the nuclear receptor transcription factor superfamily, modulates the immune microenvironment of glioblastoma, enhancing, CD8^+^ T cell antigen presentation, microglial plasticity, and the therapeutic efficacy of immune checkpoint blockade in vivo.^39^ *ZNF331*, a gene encoding a Kruppel‐associated box zinc‐finger protein, was associated with poor prognosis in oesophageal, gastric, and colorectal cancers due to gene methylation; however, its specific mechanism remains unclear.^40^, ^41^, ^42^

Subsequent GSEA enrichment analysis of the seven pivotal genes identified KEGG pathways associated with these genes. This analysis revealed a potential association between these genes, except for *CFH*, and mitochondrial metabolic reprogramming, potentially contributing to the promotion of PCa–associated bone metastasis. Lasorsa et al. demonstrated an increase in adipogenesis and cholesterol production in PCa cells, influenced by AR signalling which also controls the mixed glycolysis and oxidative phosphorylation phenotype in these cells.^43^ In benign prostate tissue, mitochondrial energy metabolism is primarily based on the oxidative phosphorylation (OXPHOS) of substrates such as glutamate and malate. However, in PCa, OXPHOS mainly relies on succinate. Furthermore, the severity of PCa positively correlates with the activity of the OXPHOS pathway.^44^ Thus, compared to hormone‐sensitive PCa, castration‐resistant PCa exhibits heightened activity of the OXPHOS pathway, suggesting a regulatory role of OXPHOS in mitochondrial metabolism that may promote the progression of PCa. Given these findings, metabolic reprogramming has emerged as a promising target for treating PCa.^45^ Our research identified a higher enrichment of *APOC1, LGALS1, NUSAP1, NR4A2, ADRB2* and *ZNF331* in the OXPHOS pathway, in PCa with bone metastasis, suggesting that targeting these genes could potentially reverse aberrant metabolic pathways and improve treatment outcomes. Further investigation is warranted understand their underlying mechanisms.

Next, we analysed immune cell infiltration in patients with PCa and with or without bone metastasis, finding a positive correlation between *CFH, APOC1* and *LGALS1* and various immune cell types. Conversely, *NR4A2*, *NUSAP1, ADRB2* and *ZNF331* negatively correlated with immune cells such as natural killer cells, monocytes and plasmacytoid dendritic cells. The potential mechanism by which CX3CR1 influences CD14^+^ CD16^−^ monocytes to facilitate the progression of PCa is through activation of the CX3CR1/CX3CL1 signalling pathway, promoting tumour angiogenesis, migration, and invasion.^9^ Given the role of monocytes in PCa cell invasiveness,^46^ and the impact of dendritic cell infiltration on patient outcomes. The FDA approved sipuleucel‐T for PCa treatment.^47^ Consequently, our findings suggest that the identified genes may serve as potential immunotherapy targets in PCa.

We conducted drug prediction and intercellular communication analysis, identifying aflatoxin B1 and benzo(a)pyrene as potent carcinogens involved in PCa development.^48^, ^49^ The preventive and therapeutic effects of Cucumis sativus (Cucurbitaceae) seed oil and indole‐3‐carbinol (I3C) against PCa induced by these chemicals have been previously reported. Furthermore, cyclosporine may be a viable treatment for hormone‐negative PCa and CRPC.^50^ In our intercellular communication analysis, we observed frequent crosstalk between osteoblasts, endothelial cells, and pericytes in PCa with bone metastasis. A close association between endothelial cells, pericytes, angiogenesis regulation, and tumour matrix remodelling within the tumour microenvironment of PCa has ben previously reported.^51^ Additionally, aging osteoblasts in osteoporosis have been shown to promote vascular endothelial cell senescence and apoptosis through exosome‐mediated signalling.^52^ However, the precise mechanisms underlying the extensive cellular communication among osteoblasts, endothelial cells, and pericytes in bone metastasis of PCa are not fully understood, necessitating further research to clarify their roles.

Next, we investigated the association between hub genes and clinical characteristics of PCa using clinical data on PFI in PCa from the TCGA database, due to the lack of other specific data for patients with bone metastasis. Our analysis revealed a significant correlation between *APOC1* expression levels and various clinical features associated with PCa progression, including PFI, pathological stage, primary treatment outcome, tumour residue, Gleason score, and lymph node metastasis, indirectly suggesting a close relationship between *APOC1* and PCa metastasis. Furthermore, our in vitro study demonstrated that silencing *APOC1* significantly reduced proliferation, clonality, migration and invasion of PCa cells, indicating that *APOC1* could be a therapeutic target for PCa with bone metastasis; however, further research is required to elucidate the specific mechanisms involved.

In summary, we employed machine learning to comprehensively analyse bulk RNA‐seq and scRNA‐seq data from primary PCa and bone metastasis, identifying seven critical to bone metastasis. We preliminarily investigated their roles in this process, correlated them with clinical features, and validated them through histological and in vitro experiments. However, our study has some limitations: (1) it used a small number of immunohistochemical samples from the same patient for both primary cancer and bone metastasis tissues; (2) the GEO database lacks comprehensive clinical and survival data for patients with bone metastases, while the TCGA database includes only a limited number of patients with metastasis (*n* = 4). Therefore, conducting a more in‐depth analysis of these genes is currently not feasible; (3) the mechanisms of these genes are not fully understood, necessitating further cell and animal experiments for verification.

## 5 CONCLUSIONS

We employed ML to analyse scRNA‐seq and bulk RNA‐seq data, identifying seven hub genes associated with bone metastasis in PCa. Their roles were further validated through immunohistochemistry, correlation with clinical characteristics of PCa, and in vitro experiments. Compared to traditional statistical methods, machine learning has the capability to handle more complex data patterns and provide more precise and comprehensive insights.^12^ In the future, multidimensional data related to PCa with bone metastasis, including genomics, epigenetics, transcriptomics, proteomics, metabolomics, can be integrated through ML, which will help to gain a deeper understanding of tumour biology and unravelling tumour plasticity and heterogeneity.^53^, ^54^

## AUTHOR CONTRIBUTIONS

**Haiyang Jiang:** Conceptualization (equal); formal analysis (lead); methodology (equal); writing – original draft (equal); writing – review and editing (equal). **Mingcheng Liu:** Data curation (equal); formal analysis (equal); software (equal); writing – original draft (equal). **Yingfei Deng:** Data curation (equal); formal analysis (equal); software (equal); writing – original draft (equal). **Chongjian Zhang:** Formal analysis (equal); methodology (equal). **Longguo Dai:** Data curation (equal); formal analysis (equal). **Bingyu Zhu:** Data curation (equal); methodology (equal). **Yitian Ou:** Investigation (equal); software (equal). **Yong Zhu:** Data curation (equal); software (equal). **Chen Hu:** Data curation (equal); resources (equal); visualization (equal). **Libo Yang:** Resources (equal); supervision (equal). **Jun Li:** Supervision (equal); writing – review and editing (equal). **Yu Bai:** Resources (equal); supervision (equal); writing – review and editing (equal). **Delin Yang:** Supervision (equal); validation (equal); writing – review and editing (equal).

## FUNDING INFORMATION

This research was supported by the funding of the National Natural Science Foundation of China (No 82160511); National Cancer Center Climbing Fund (No NCC201925B01); The applied basic research project of Yunnan province‐Kunming Medical University joint fund (202101AY070001‐160); Scientific Research Fund of Education Department of Yunnan Province (2022J0246).

## CONFLICT OF INTEREST STATEMENT

The authors declare that they have no competing interests.

## CONSENT

Informed consent was obtained from all subjects involved in the study.

## INSTITUTIONAL REVIEW BOARD STATEMENT

This study was approved by the ethics committee of The Third Affiliated Hospital of Kunming Medical University (Peking University Cancer Hospital Yunnan, Yunnan Cancer Hospital, Cancer Center of Yunnan Province) with approval number KYLX2024‐032 and carried out under the World Medical Association Declaration of Helsinki.

## Supporting information


Figure S1.

Figure S2.

Figure S3.

Figure S4.

Figure S5.

Figure S6.

Table S1.

## Data Availability

The authors are able to provide the data generated by the analysis of this study upon reasonable request.
