# Supplementary figures and images for: Identification of prostate cancer bone metastasis related genes and potential therapy targets by bioinformatics and in vitro experiments
Source: J Cell Mol Med. 2024 Aug 4;28(15):e18511. doi: 10.1111/jcmm.18511 (PMC11298316; doi:10.1111/jcmm.18511)

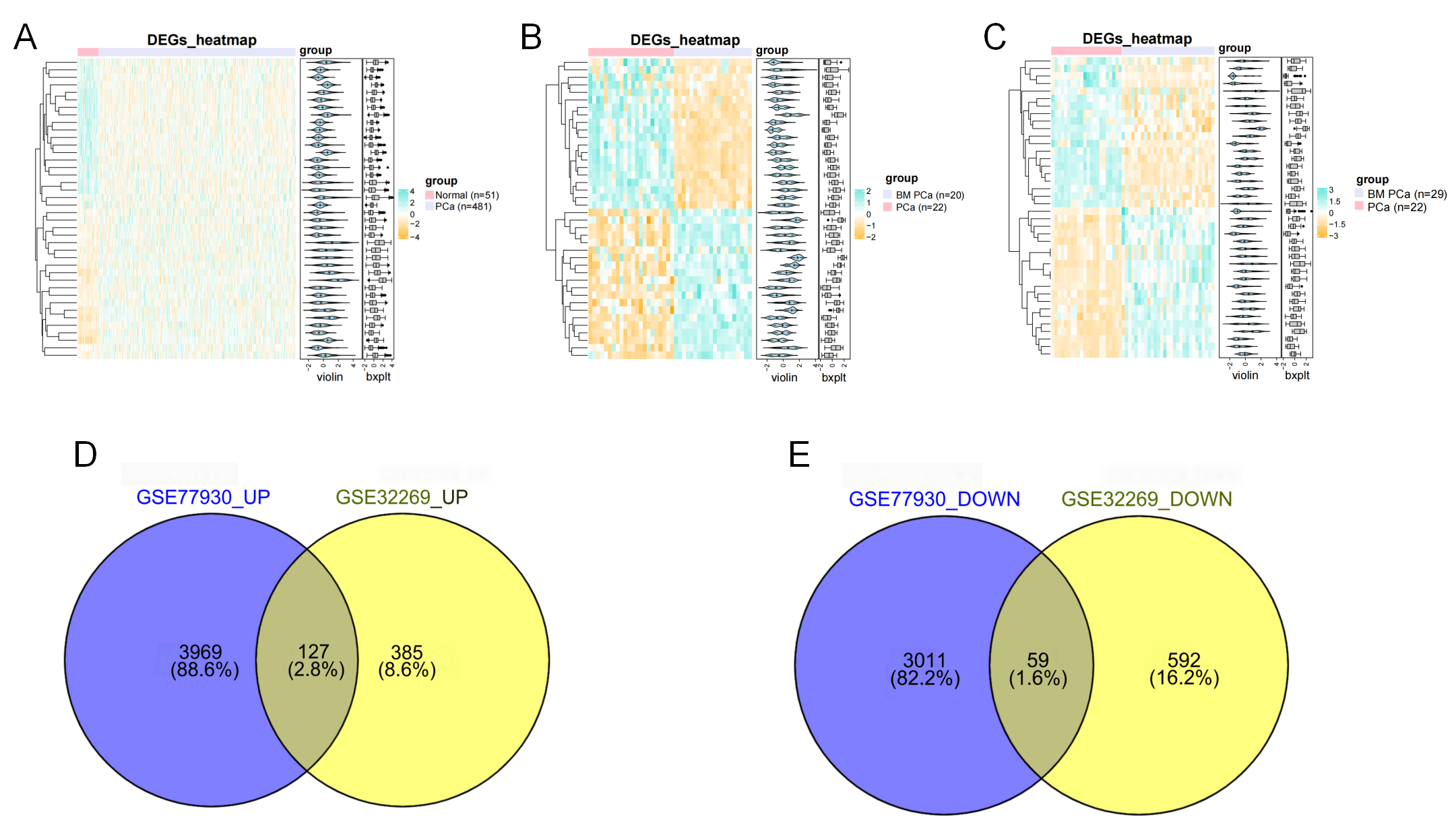

Supplement: Supplementary file 1 — Figure S1. [file JCMM-28-e18511-s002.png]

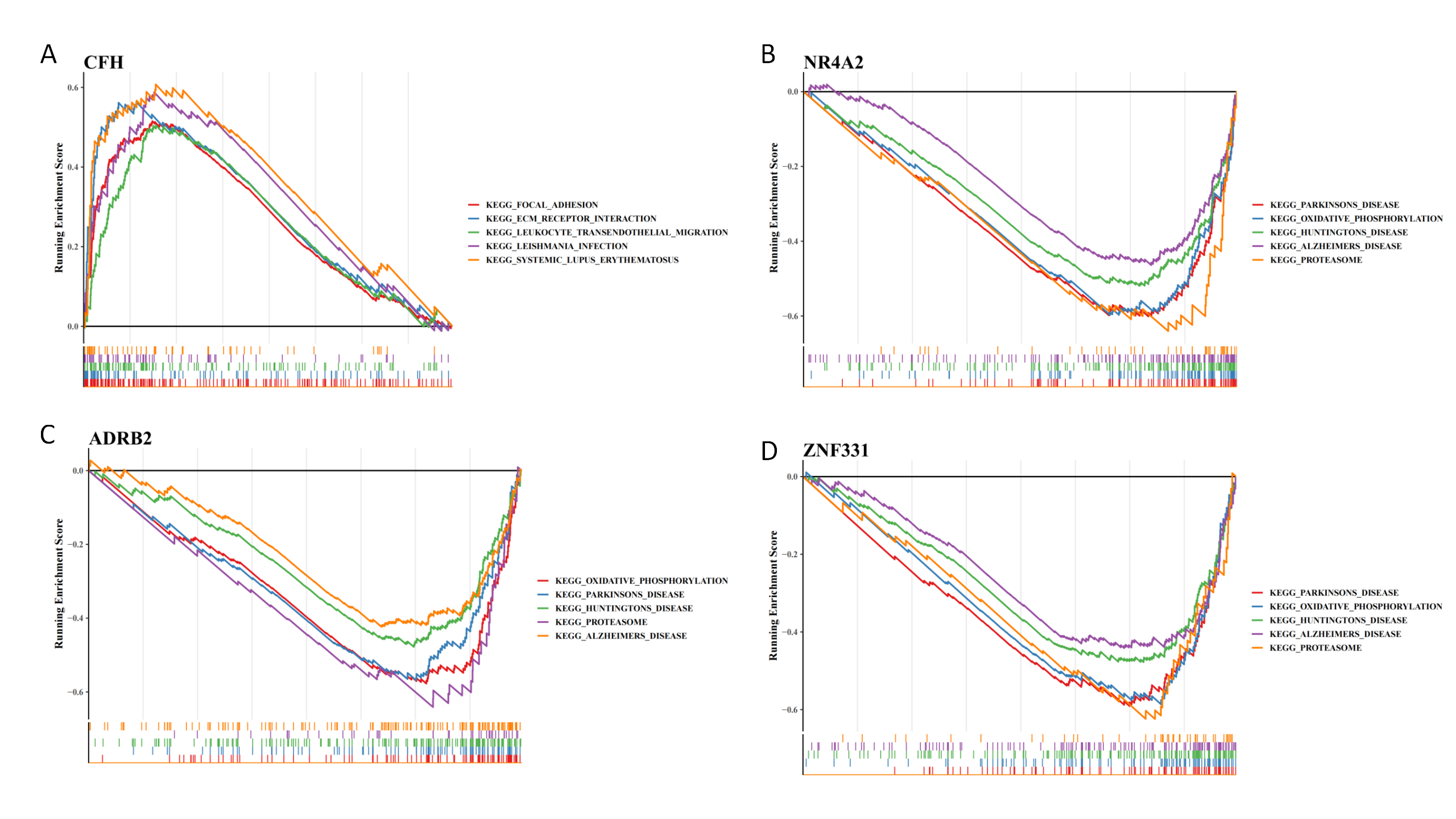

Supplement: Supplementary file 2 — Figure S2. [file JCMM-28-e18511-s001.png]

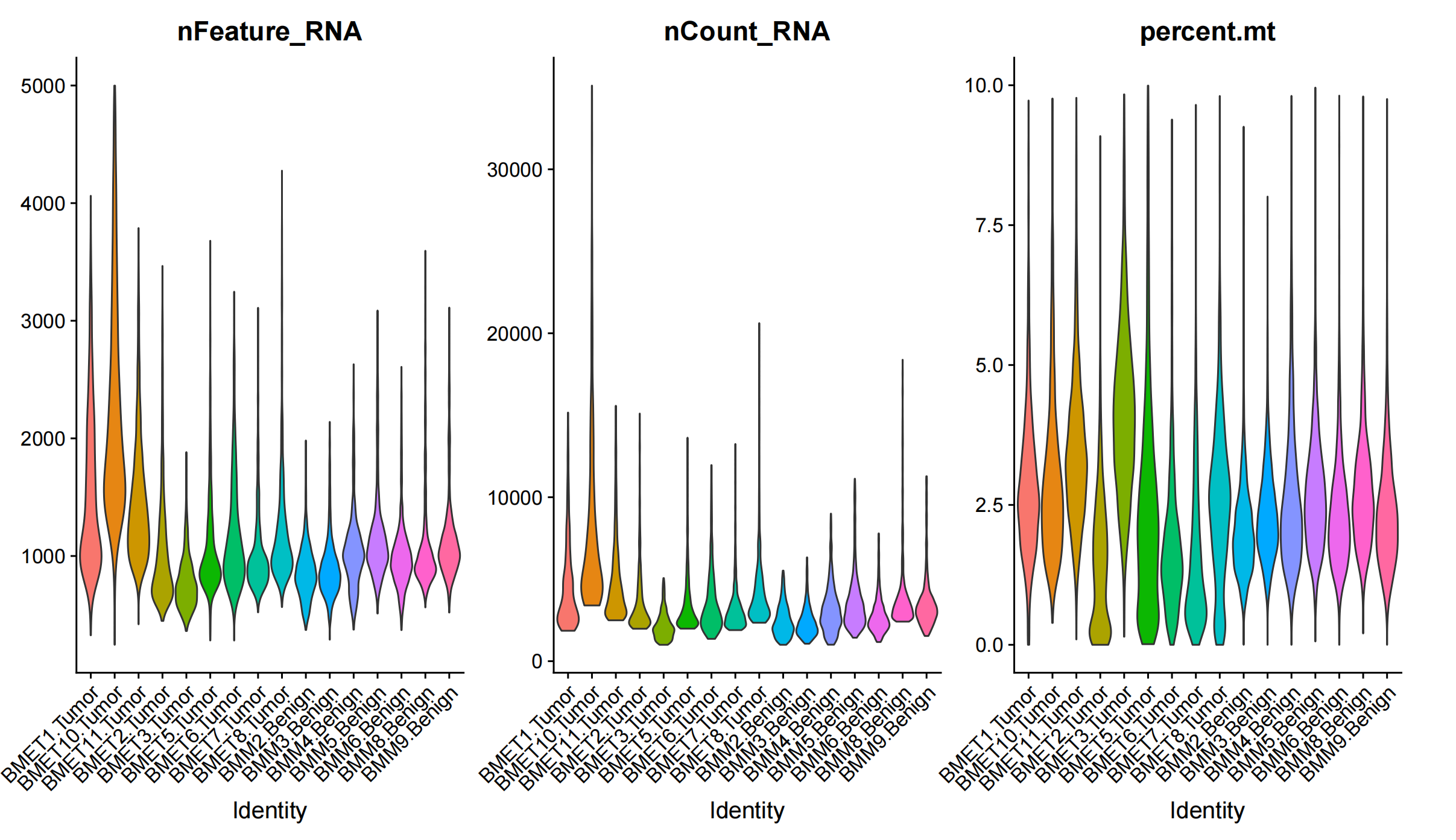

Supplement: Supplementary file 3 — Figure S3. [file JCMM-28-e18511-s005.png]

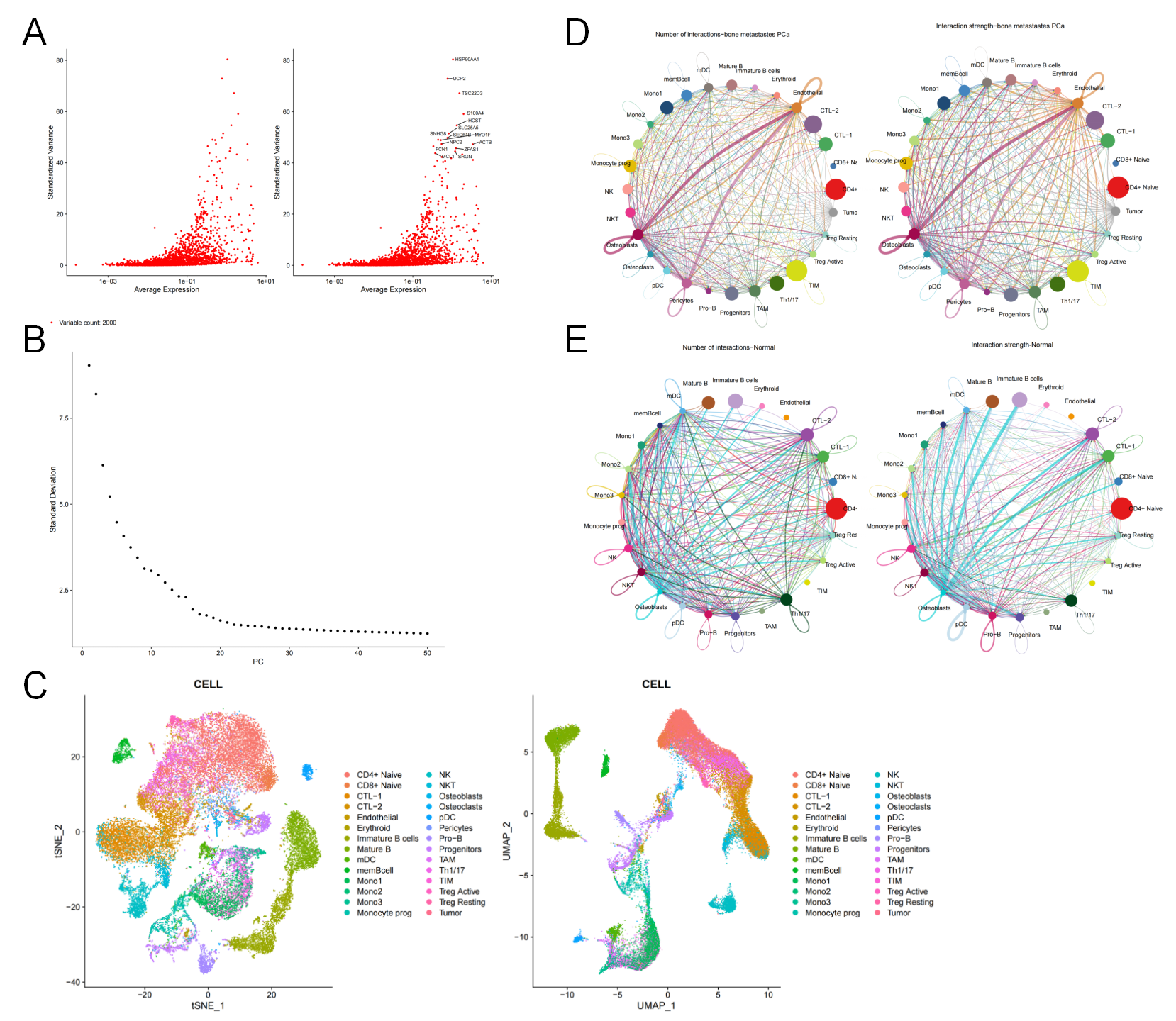

Supplement: Supplementary file 4 — Figure S4. [file JCMM-28-e18511-s003.png]

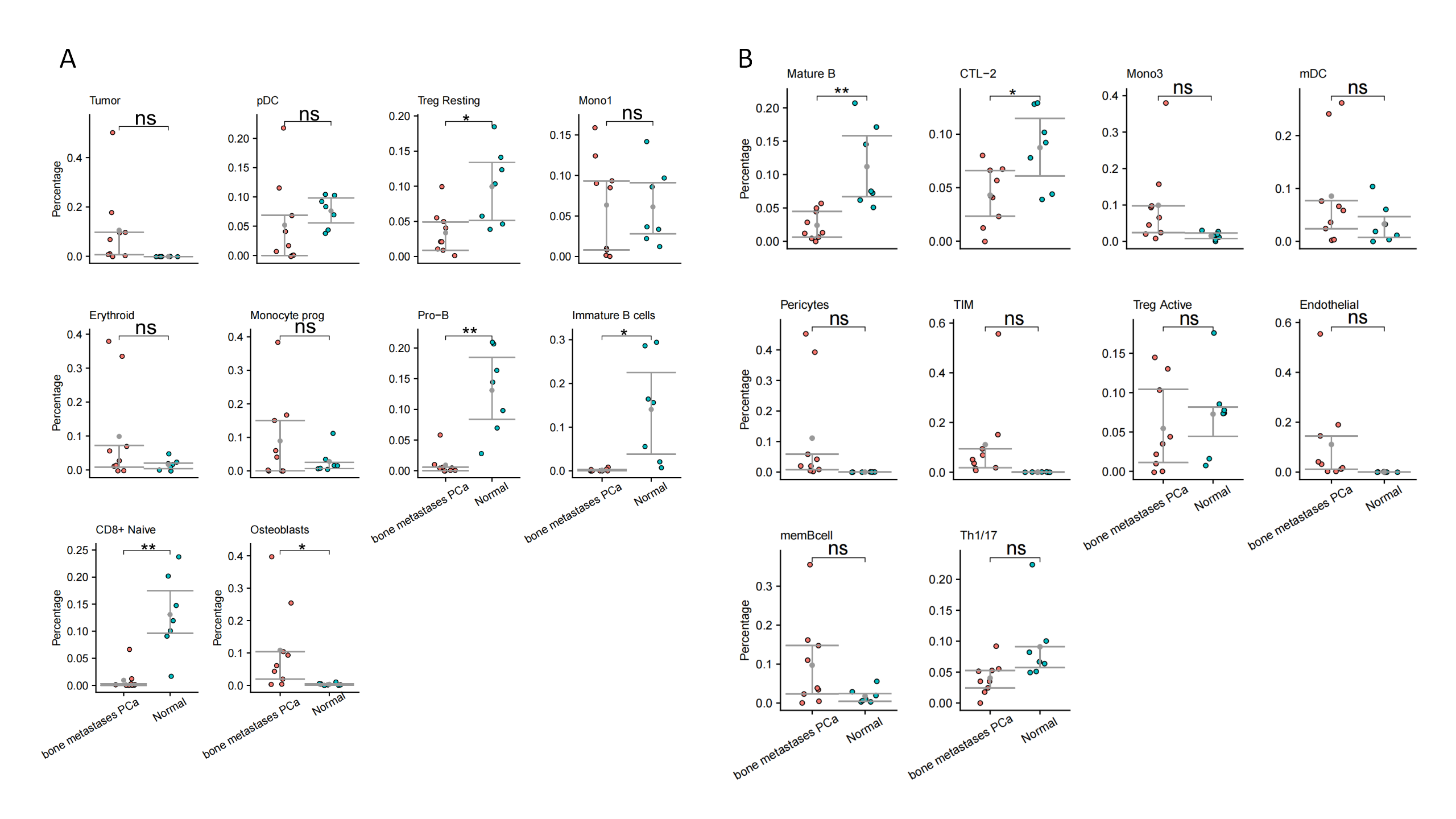

Supplement: Supplementary file 5 — Figure S5. [file JCMM-28-e18511-s007.png]

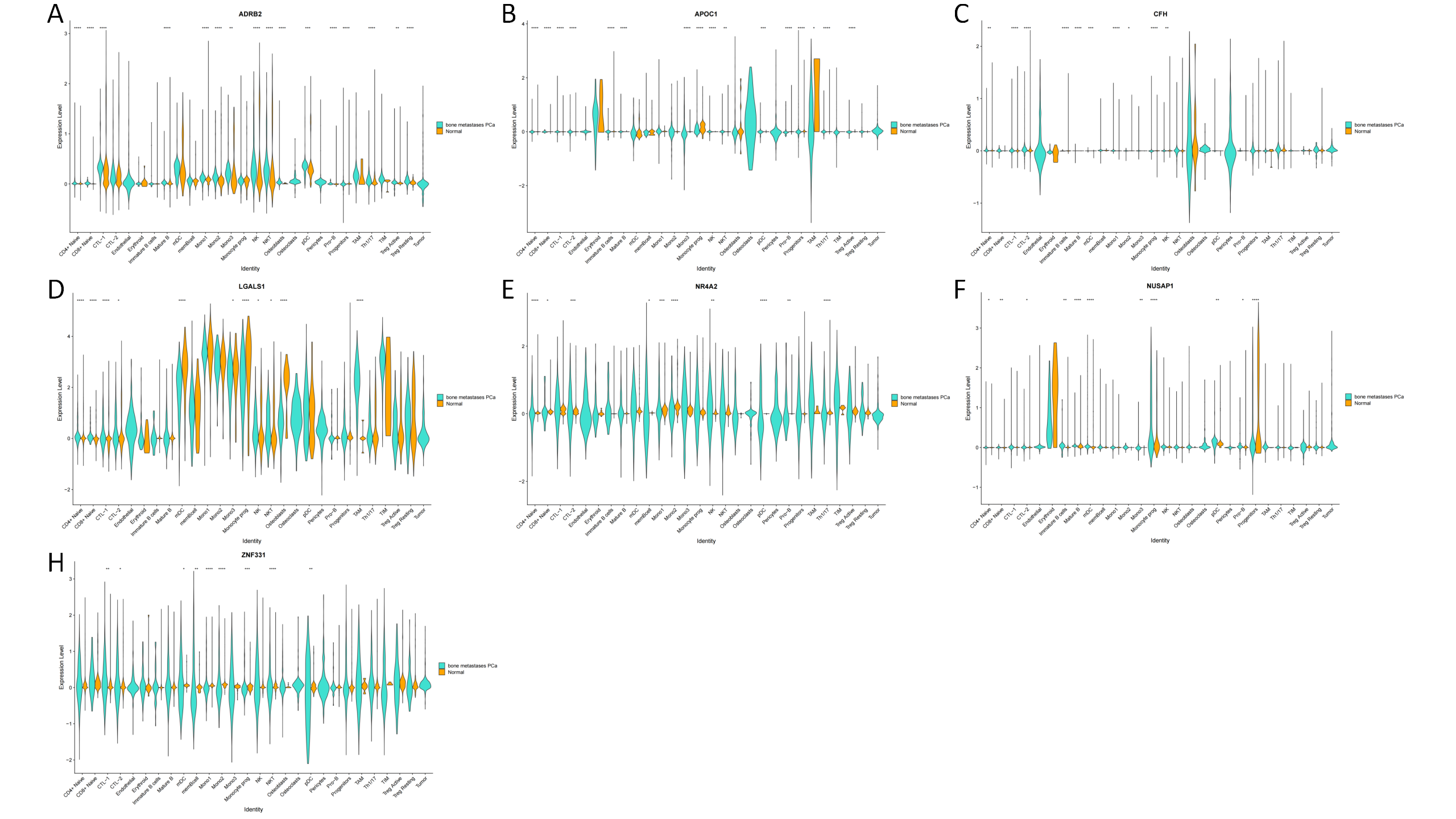

Supplement: Supplementary file 6 — Figure S6. [file JCMM-28-e18511-s006.png]
